# Carbon Monoxide Stimulates Chondrocyte Mitochondria and Protects Mitochondria During Cartilage Injury

**DOI:** 10.3390/antiox14050514

**Published:** 2025-04-25

**Authors:** Suryamin Liman, Madeline R. Hines, Piedad C. Gómez-Contreras, Emily Witt, Jacob S. Fisher, Kevin J. Lu, Lauren D. McNally, Alicia T. Cotoia, Maxwell Y. Sakyi, Brett A. Wagner, Michael S. Tift, Douglas Fredericks, Jessica E. Goetz, James D. Byrne, Mitchell C. Coleman

**Affiliations:** 1 Department of Radiation Oncology, University of Iowa, Iowa City, IA 52242, USA; suryamin-liman@uiowa.edu (S.L.); piedad-contreras@uiowa.edu (P.C.G.-C.); emily-witt-1@uiowa.edu (E.W.); brett-wagner@uiowa.edu (B.A.W.);; 2 Department of Orthopedics and Rehabilitation, University of Iowa, Iowa City, IA 52242, USA; madeline-hines@uiowa.edu (M.R.H.); jacob-fisher@uiowa.edu (J.S.F.); kevin-lu-1@uiowa.edu (K.J.L.); lauren-mcnally@uiowa.edu (L.D.M.); maxwell-sakyi@uiowa.edu (M.Y.S.); douglas-fredericks@uiowa.edu (D.F.); jessica-goetz@uiowa.edu (J.E.G.); 3 Department of Biology and Marine Biology, University of North Carolina Wilmington, Wilmington, NC 28403, USA; atc5878@uncw.edu (A.T.C.); tiftm@uncw.edu (M.S.T.)

**Keywords:** cartilage, carbon monoxide, mitochondria, glutathione, monochlorobimane

## Abstract

***Objective:*** Joint injury precipitates post-traumatic osteoarthritis (PTOA) via chondrocyte mitochondrial oxidative damage. Carbon monoxide (CO) is a small molecule with potent antioxidant and mitochondrial benefits in other tissues that have not been explored in healthy chondrocytes. We hypothesized that CO would subvert the mitochondrial effects of articular cartilage injuries upon resident chondrocytes. ***Design:*** We evaluated intra-articular delivery of a novel carbon monoxide-containing foam (COF). We used in vitro impact injuries to explore mitochondrial and redox endpoints after CO exposure. We then applied intra-articular injections of COF or control room air foam (RAF) to assess safety, efficacy, and other intra-articular responses. ***Results:*** COF increased the expression of HO1 and mitofusin-1 within 1 h and this increase was sustained for 12 h in vitro. COF increased chondrocyte mitochondrial respiration by 40% and increased reduced (not oxidized) thiols by 50% following in vitro injury to osteochondral explants. After cartilage injury, COF prevented the formation of 3-nitrotyrosine and the loss of articular chondrocyte mitochondria. When injected intra-articularly, COF was retained for 24 h post-injection in mouse stifle joints. It increased HO1 in those joints, enhanced reduced thiol levels in rabbit stifle joints, and exhibited no toxicity 1 and 4 weeks after injection. ***Conclusions:*** This study supports the hypothesis that CO functions as an antioxidant for articular chondrocytes by supporting mitochondria and intracellular GSH in the presence or absence of cartilage injury. Challenges in delivering exogenous CO have limited its preclinical development, but new CO-releasing materials like COF may enable new examinations of this promising small molecule.

## 1. Introduction

PTOA is a debilitating joint disease that can develop after a joint injury. Injuries to articular cartilage are known to initiate oxidative damage to resident articular chondrocyte mitochondria over the first few hours after injury [1,2]. Similar damage to chondrocytes can result from intra-articular surgical exposures, whether from exposure to air [3,4,5], low osmolarity [6,7], unbuffered pH [8], or iatrogenic injuries [9]. This led to our working preclinical hypothesis that applying protective strategies prior to intra-articular surgery might improve the efficacy or success of those surgeries. We have shown protection from cartilage injuries, with N-acetylcysteine-mediated increases in intracellular glutathione (GSH) or the inhibition of mitochondrial electron transport, but these non-specific approaches only provided partial protection from PTOA after intra-articular fracture [10,11,12]. This study investigates a new approach to protecting articular cartilage through the coordinated manipulation of chondrocyte mitochondrial and GSH pathways.

When considering small molecules that might provide the necessary mitochondrial benefits while supporting GSH, we became interested in exploring the effects of carbon monoxide (CO). This endogenously produced gasotransmitter broadly binds to heme-containing proteins, inhibiting them and upregulating heme oxygenase-1 (HO1) [12,13,14,15,16,17,18,19]. This leads to the restoration of redox balance, mitochondrial function, and energetic pathways in mammalian cells following injury [20,21,22,23,24,25]. CO donors [16,26] and the transgenic upregulation of HO1 have demonstrated some degree of protection against inflammatory arthritis and decrease inflammatory readouts in arthritic cartilage [27,28,29] as well as a wide variety of non-orthopedic pathologies [30,31,32,33,34,35,36]; however, the direct mitochondrial and redox effects of CO on normal cartilage and chondrocytes have not been described and prior examinations relied upon CO donors rather than the direct administration of CO gas. The response of otherwise healthy tissues is an important consideration for intra-articular delivery, therapeutic outcomes, and safety. Here, we hypothesized that CO would improve mitochondrial metabolism while decreasing oxidation in chondrocytes after cartilage injury without demonstrating any adverse effects in vitro or in vivo.

To examine the potential benefits of CO, we opted to use CO foam (COF) [31] which is capable of being injected intra-articularly. We investigated the application of COF to chondrocytes in monolayer culture, primary bovine osteochondral explants, and rabbit and mouse stifle (quadruped knee) joints. We demonstrate that, after cartilage injury, CO can improve GSH and increase mitochondrial activity in chondrocytes in vitro, and that the intra-articular application of COF replicates these effects in situ.

## 2. Materials and Methods

### 2.1. Application of CO

Intact bovine stifle (knee) joints obtained from a local abattoir (Bud’s Custom Meats, Riverside, IA, USA) were dissected approximately 2 h after death. Fresh, healthy cylindrical bovine osteochondral explants 10 mm in diameter were obtained from the load-bearing region of both the medial and lateral femoral condyles. Explants were washed in Hank’s balanced salt solution (HBBS) with 50 U/mL penicillin, 50 µg/mL streptomycin, and 2.5 µm/mL amphotericin B. They were then placed into culture medium (45% Dulbecco’s Modified Eagle’s medium, 45% F-12, 10% fetal bovine serum (FBS) (all Gibco, Waltham, MA, USA), 100 U/mL penicillin, 100 µg/mL streptomycin, and 2.5 µm/mL amphotericin B). All specimens were kept in a humidified tissue culture incubator maintained at 5% O_2_, 5% CO_2_, and 37 °C. Explants were equilibrated to culture conditions overnight before any treatment or testing.

To generate the COF and control foams (room air (RAF), nitrogen (NF), and 5% O_2_), we dissolved xanthan gum (0.5 weight (wt) %, Modernist Pantry, Eliot, ME, USA) and methylcellulose (0.8 wt %, Modernist Pantry, Eliot, ME, USA) in phosphate-buffered saline (PBS) to form a pre-foam solution. The pre-foam solution was then inserted into a custom-made whipping siphon and pressurized to 200 PSI with each gas of interest according to Byrne et al. [31,35]. Foams containing each gas were filled into 15 mL conical tubes, and the explants submerged in the foam for 60 min. An explant cultured in media as normal and not exposed to any foam, was used as a no-foam or sham control.

### 2.2. Induction of HO1 by CO

For this experiment, we utilized COF as well as a control foam containing 5% oxygen-like incubation conditions to minimally disturb control cartilage oxygen tension during the determination of HO1 responses. After exposure to the foam, the explants were rinsed with culture media equilibrated to incubator conditions. For Western blotting, the explants were harvested 0, 1, 3, 6, or 12 h after the initial 60 min exposure to either foam. The cartilage was minced and mixed with RIPA buffer (Invitrogen, Waltham, MA, USA) and placed into −80 °C freezer. Protein extracts from explants specimens were denatured and reduced by the addition of LDS sample buffer (Invitrogen, Waltham, MA, USA) and DTT reducing agent (Invitrogen, Waltham, MA, USA), heated at 70 °C for 10 min. A total of 20 ug of protein was loaded per well and electrophoresed through a 10% Bis-Tris NuPage acrylamide gel (Invitrogen, Waltham, MA, USA) at a 120-volt constant for 2 h with MES-SDS running buffer (Invitrogen, Waltham, MA, USA). After electrophoresis, the gel proteins were transferred to a 0.2 uM PVDF membrane (BioRad, Hercules, CA, USA) at 300 mAmp constant for 2 h with 20% methanol 0.1% SDS transfer buffer (Invitrogen, Waltham, MA, USA). The blotted membrane was stained using Ponceau S, as a loading and protein quality control. The membrane was blocked for 30 min with bovine serum albumin (BSA) 5% solution. The primary antibodies HO1 (Cell Signaling, Danvers, MA, USA) and mitofusin-1 (Abcam, Cambridge, UK) were diluted 1:1000 in BSA 2.5% and incubated overnight at 4 °C in a rotator shaker. After TBST rinsing, HRP-linked secondary antibody (Cell Signaling, Danvers, MA, USA), diluted 1:2000 in BSA 2.5%, was incubated by 1 h RT. After TBST rinsing, the protein signal was developed by Super Signal West Femto (Thermo, Waltham, MA, USA) and visualized with Amersham Hyperfilm ECL system (GE Healthcare, Chicago, IL, USA) film. Densitometry was performed in ImageJ Fiji 2.16.0 using the actin staining as a standardized control and represents the mean of *n* = 3.

### 2.3. Chondrocyte Extracellular Flux Analyses

Primary bovine chondrocytes were extracted from articular cartilage and used to evaluate mitochondrial activity in live chondrocytes immediately after COF or room air-containing foam (RAF) exposure. Briefly, cartilage from the explants was digested with collagenase and pronase (0.1 mg/mL; Sigma-Aldrich, St. Louis, MO, USA) overnight. Chondrocytes were then pelleted, resuspended, and plated. Chondrocytes were cultured as described above for explants. Then, 20,000 primary bovine chondrocytes per well were plated on XF96 Extracellular Flux Analyzer (Seahorse Bioscience, Agilent, Santa Clara, CA, USA) plates. After allowing 3 days for attachment, the wells were exposed to either RAF or COF for 30 min. Media were then changed and a mitochondrial stress test was conducted as soon as possible after foam treatment, as previously described [10]. Briefly, we used the following successive injections into each well using concentrations previously determined to be effective and non-lethal in primary chondrocytes (final concentration in the well is shown): oligomycin (2 μM), carbonyl cyanide-4-(trifluoromethoxy) phenylhydrazone (FCCP) (250 nM), antimycin A (5 μM), and rotenone (2 μM). Mitochondrial stress test measurements were obtained approximately 1.5 h after initial exposure, following extracellular flux equilibration and early portions of the mitochondrial stress test. After completing the assay, the cells were trypsinized in 100 μL of trypsin. After cells were lifted from plates, 150 μL of media was used to neutralize the trypsin and cells were counted using a hemocytometer. Oxygen consumption rates (OCRs) were normalized to the number of cells. Basal respiration is reported as basal OCR minus OCR after rotenone and antimycin A injection. ATP-linked respiration is reported as the basal OCR minus OCR after oligomycin injection. Proton leakage is the OCR after oligomycin injection minus the OCR after rotenone and antimycin A injection, normalized to each specimen’s basal respiration. The mitochondrial data were statistically compared with a Student’s *t* test. Statistical significance was defined as a *p*-value of less than 0.05.

### 2.4. Live Cell Evaluation of ATP and Mitochondrial Content After Gaseous Manipultaion

To determine the effect of gaseous manipulations on articular chondrocyte ATP abundance, osteochondral explants were exposed to RAF or COF for 1 h then returned to 5% O_2_ pre-gassed media and cultured for 24 h. Live cell stains were prepared in DMEM/F-12 media without phenol red (Gibco, Waltham, MA, USA). BioTracker ATP-Red Live Cell Dye (Sigma, #SCT045, St. Louis, MO, USA) was diluted to 5 µM and co-stained BioTracker 405 Blue Mitochondria Dye (Sigma, #SCT135, St. Louis, MO, USA) at a concentration of 200 nM for 15 min. Images were gathered using a confocal microscope (Olympus/Evident, Tokyo, Japan) with a 20× immersion objective using wavelengths (450 nm, DAPI, respectively). Three images were taken from each sample for analysis. Images were analyzed in the red channel using ImageJ Fiji 2.16.0 to determine the mean intensity and the area of the image. All quantitative measurements were graphed, and statistical analysis was performed in Graphpad Prism 9.

### 2.5. Mechanical Injury and Evaluation of Thiol Redox Status

The effect of CO on chondrocytes receiving direct mechanical injury was assessed by applying foams and subjecting explants to well-characterized, energy-controlled impact injuries similar to prior studies [1,10,11,12]. This model creates a simple and highly reproducible impact injury with cell biological features comparable to those of in vivo traumatic joint injuries [12]. After overnight equilibration to culture conditions, explants were treated with either sham, RAF, COF, or NF for 1 h. Following treatment, the explants were rinsed with media. Explants were adhered to a 3 × 3 cm stainless steel plate with polycaprolactone. The cartilage was then impacted with a flat, stainless steel impermeable platen via a drop tower that delivered an energy-controlled, gravity-driven 2 J/cm^2^ [1,10,11,12]. The explants were rinsed with HBSS before being returned to the normal cell culture medium for 24 h. Four explants were used in each group unless otherwise noted.

To assess the redox status and cell viability of chondrocytes in situ, we used confocal microscopy of monochlorobimane (MCB) (Invitrogen, Waltham, MA, USA) to provide detailed views of intracellular thiol status throughout a cross-section of the articular surface. First, explants were cut through the center of the impacted region (or equivalent in non-impacted control samples) with a precision saw (IsoMet 1000, Buehler, Lake Bluff, IL, USA). Next, a scalpel was used to cut away a full-thickness cross-section, i.e., from the articular surface to the subchondral bone, excising a 0.25 mm layer of cartilage to remove the tissue abraded by the saw, thus exposing an undamaged surface. Explants were stained in DMEM/F-12 media without phenol red (Gibco, Waltham, MA, USA) containing MCB (20 μM) and Calcein AM (1 μM, Invitrogen, Waltham, MA, USA), to assess cellular viability. MCB reacts with endogenous glutathione-S-transferase and reduced thiols (predominantly GSH) to create a blue, fluorescent complex. This signal is not entirely specific to GSH but is dominated by GSH, as shown previously [37,38]. Thus, blue fluorescence indicates the presence of reduced thiols and decreases in blue fluorescence would indicate oxidation. We previously showed that GSH is a critically important mediator of chondrocyte injury by using Griffith’s biochemical GSH/GSSG assay on whole cartilage [39], but MCB allows the visualization of GSH status in space on a per cell basis [40]. This is important for testing the application of a diffusible, gaseous therapy like CO to a thick, architecturally complex tissue like cartilage. The samples were then visualized with a confocal microscopy (Olympus/Evident, Tokyo, Japan), using excitations at 405 nm and 488 nm for MCB and Calcein AM, respectively.

Three micrographs of each impacted area from each specimen were exported and manually segmented into regions of interest. Here, we report data for the superficial zone, where the effects of each foam and impact are clearly apparent. MCB intensity was analyzed with a previously published, custom MATLAB (version 2024)-based algorithm [40]. This algorithm uses a watershed-based algorithm to identify individual calcein AM-stained live chondrocytes (several hundred per micrograph), and then only quantifies intracellular MCB fluorescent intensity in each live cell while discarding data from any dead cells in the image. The average intensity of intracellular MCB staining was compared between foam-treated and injury and non-injury groups then analyzed using two-way ANOVA with Sidak’s multiple comparisons test. Normality was confirmed using a Shapiro–Wilk test, yielding a *p*-value of 0.4673.

To support that MCB measurements are related to GSH specifically, we also utilized Griffith’s GSH/GSSG assay [39]. Cartilage samples cut from explants were minced into 5% sulfosalicylic acid and subjected to three freeze–thaw cycles to lyse the cells. Tissue and cell lysates were then mixed with buffer solution containing dithionitrobenzoic acid (DTNB), GSH reductase, and NADPH (all from Sigma-Aldrich, St. Louis, MO, USA). GSH reacts with DTNB to form a yellow product, while the GSH reductase recycles the GSH. As a result, the absorbance rate change at 412 nm as measured via a spectrophotometer is proportional to GSH concentration. All sample concentrations were determined via comparison to appropriate standard curves. For GSSG determination, a 20% sample volume of 2-vinylpyridine (Sigma-Aldrich, St. Louis, MO, USA) was added to sample aliquots for 1 h before analysis to quench GSH and prevent the reaction of reduced GSH with DTNB.

### 2.6. Immunofluorescent Evaluation of Mitochondira and Oxidative Damage

To evaluate mitochondrial content and oxidative damage in tissue after gaseous manipulation and to assess impact, articular cartilage was evaluated for the mitochondrial marker translocase of outer mitochondrial membrane (TOMM20) and for oxidative damage marker 3-nitrotyrosine (3NT). Following the foam and impact assessment and the bisection of the osteochondral explants, one of the halves from each group was placed 10% neutral-buffered formalin with 0.03% Safranin-O (Saf-O). In brief, the samples were decalcified, processed, paraffin-embedded, and sectioned to 5 µm onto super frost slides. The slides were stained on the Discovery Ultra (Roche, Basel, Switzerland) using either rabbit primary anti-TOMM20 (1:50 Cell Signaling, #42406) or anti-nitrotyrsoine (1:150, Millipore, St. Louis, MO, USA). The fluorescent secondary antibody was goat anti-rabbit cy5 (Roche, 760-238, Basel, Switzerland). The slides were imaged using the Olympus VS200. Images were analyzed by selecting 3 sites on the edges and center of the impact site or similar regions on unimpacted samples. Analysis was manually segmented to cover the superficial and transitional zone of cartilage (the first 200 µm from the articular surface). The mean intensity and area were measured for each image in ImageJ Fiji 2.16.0. The average mean intensity/area was calculated and graphed and statistical analysis was performed in Graphpad Prism 9.

### 2.7. Intra-Articular COF and Evaluation In Vivo

All animal studies were conducted under the review and approval of the Institutional Animal Care and Use Committee at the University of Iowa [Approval #3062034, approved 19 July 2023] and all associated regulations were followed. To first demonstrate that the intra-articular injection of COF could effectively and efficiently fill the joint space, we administered up to 4 mL of COF containing the contrast agent Isovue-370 into the medial compartments of the stifles joints of 8- to 9-month-old female New Zealand White rabbits (Charles River, Wilmington, MA, USA) immediately post-mortem. The COF was injected through the stifle joint via a 27 Ga needle placed immediately medial to the patellar tendon. During injections, we observed no backflow out of injection sites, supporting reliable intra-articular delivery. The presence of foam within the joint was confirmed via the incorporation of 20% Isovue-370 in the foam solution followed by fluoroscopic imaging. The same contrast agent-containing foam was also used to assess the retention of COF in murine stifle joints.

To investigate whether the intra-articular injection of COF has similar effects to in vitro experimentation showing increased intracellular GSH or reduced thiols, we utilized the RAF and COF stifle joints, as described above. The stifle joints were then disarticulated, rinsed in culture media, and equilibrated in the same media and conditions overnight as explants for comparison. This was followed by MCB staining of the articular surfaces 24 h later. The lateral femoral condyles were imaged at the center of the articular surface for live cell imaging using MCB and calcein AM, as described above. The MCB intensity was compared with paired *t* tests.

To determine if COF increased HO1 expression in situ, post-mortem bilateral injections of either COF or RAF. The stifles were dissected and incubated for 24 h, fixed, decalcified, processed, paraffin-embedded, and sectioned at 5 µm. HO1 was evaluated using immunofluorescence. In brief, slides were placed in citrate-buffered antigen retrieval at 55 °C overnight, blocked with normal goat serum (88.5% PBS, 10% normal goat serum, 1% bovine serum albumin, 1% cold water fish gelatin 9% in PBS, and 0.05% Tween 20, followed by overnight primary antibody rabbit anti-HO1 (1:150, Cell Signaling, Danvers, MA, USA) incubation at 4 °C. The samples were rinsed followed by 30 min incubation of the goat anti-rabbit Alexa Cy5 (1:500, Abcam, #AB656, Cambridge, UK) fluorescent antibody, followed by DAPI mounting media and cover slipping. The slides were images using a confocal microscope (Olympus/Evident, Tokyo, Japan) at 20× wavelengths (Cy5, and DAPI).

To investigate the safety and toxicity of COF for normal synovium and cartilage, NZW rabbits were randomly distributed for 1-week and 4-week observation, with *n* = 10 (5 male, 5 female) in each observation period. We compared stifle joints from rabbits with no injection (Normal, *n* = 2) to stifles from rabbits that received a 4 mL injection of RAF in one stifle and COF in the opposing stifle, alternating on the left and right for both injections in individual animals. At harvest times of 1 week or 4 weeks after injection, articular surfaces were placed into culture media to assess chondrocyte viability, described below, while synovia were dissected from the posterior of the stifles and then pinned to cork sheets for fixation (10% NBF) and processed. After dehydration, synovia were bisected at the midline and paraffin-embedded with the cross-sectional facedown to provide full-thickness views of the synovia. Cross-sections of the synovium were stained with hematoxylin and eosin (H&E) and then synovial thickness was measured using Olympus Desktop. We next estimated the cellularity of synovia as the number of cells thick the tissue presented in these cross-sections, counting 5 random sites per synovium and then averaging these counts. To assess chondrocyte viability, we stained the tibial articular surfaces with Calcein AM (Life Technologies, Waltham, MA, USA), imaged via confocal microscope (Olympus/Evident, Tokyo, Japan) using a 10× objective to view the cartilage from above, and then counted viable cells per visual field with watershed-based ImageJ Fiji 2.16.0 macros. The cell counts were compared among groups with two-way ANOVA.

To assess CO concentrations after intra-articular injection, we utilized C57B6J mice of both sexes, aged 8 to 15 weeks (Jackson, Bar Harbor, ME, USA) Six mice were randomly assigned to each group (3 males and 3 females) and were given injections of 0.05 mL RAF or COF under anesthesia with a 31 Ga needle; they were then returned to their cages to move freely. Stifles were harvested 24 h later and frozen. For analysis of CO content, stifles were dissected down to the joint capsule, rinsed free of blood with PBS, and placed in 2 mL bead mill tubes with stainless steel beads (2.4 mm) and diluted 10-fold with ice cold water. Samples were then homogenized on a bead-mill homogenizer (VWR, Radnor, PA, USA), and placed in an ultrasonic bath (Branson/Emersen, St. Louis, MO, USA) for 5 min. Using a gas-tight syringe (Hamilton, Bonaduz, Switzerland), 20 µL of homogenate was then added through a septum to a CO-free amber borosilicate glass vial that contained 20 µL of sulfosalicylic acid (20%). The CO released into the headspace of the vial was then flushed through a gas chromatograph with a reducing compound photometer (Peak Labs, Moutain View, CA, USA; Peak Performer 1) and quantified using a standard curve, created daily using different volumes of 1 ppm CO gas (AirGas, Radnor, PA, USA).

## 3. Results

### 3.1. CO Rapidly Induces HO1 and Increases Mitochondrial Function

**Figure 1:**
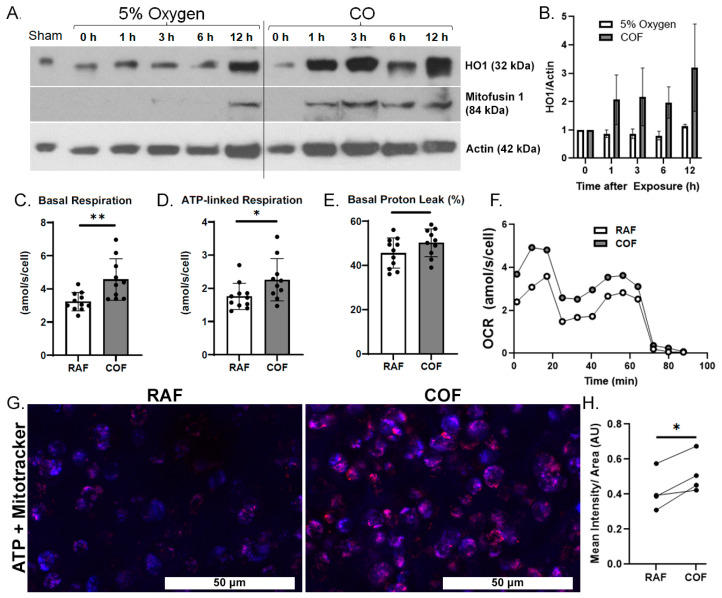
CO rapidly induces chondrocyte HO1, mitofusin-1, and mitochondrial metabolism. (**A**) Western blots of tissue homogenates from explants at various timepoints after CO exposure show increasing presence of HO1 within 1 h after CO compared to control specimens exposed to normal 5% O2 gassed solutions, with densitometric analyses shown in (**B**). This coheres with increased presence of mitofusin-1. Mitochondrial stress tests of chondrocytes extracted from similarly treated cartilage showed increased per-cell (**C**) basal respiration, (**D**) ATP-linked respiration, and no indications of damage to the mitochondrial electron transport chain, as indicated by lack of change in (**E**) proton leakage. (**F**) Mean OCR from mitochondrial stress tests. *n* = 11; * *p* < 0.05, ** *p* < 0.01, by *t* test. (**G**) Representative images of bovine articular cartilage 24 h after RAF or COF treatment stained with live cell dyes for ATP (red) and mitotracker (blue) colocalization (magenta) demonstrated that COF treatment has increased ATP-positive staining, magnification 20×. (**H**) Quantitation of ATP staining demonstrated COF staining was significantly increased compared to paired RAF controls; paired *t*-test * *p* < 0.05.

We first wanted to demonstrate whether COF induces the expression of HO1 in articular cartilage. COF increases HO1 on Western blots as soon as 1 h after exposure and this elevation lasted for at least 12 h, as shown in Figure 1A,B. We also observed a simultaneous increase in mitofusin-1, a marker of mitochondrial fusion previously observed with CO exposure as well as mitochondrial health in other tissues [41].

We then evaluated whether CO could increase the mitochondrial activity of articular chondrocytes. Live chondrocytes exposed to COF in culture showed 40% higher basal OCR and 30% more ATP-linked respiration, as indicated by mitochondrial stress tests. Results are shown in Figure 1C,D. These increases occurred without a significant difference in proton leakage, as shown in Figure 1E, suggesting no increases in oxidative stress or evidence of damage to electron transport chains from the increased mitochondrial activity. Throughout these tests, mean OCRs per cell were 30−40% greater for COF-treated chondrocytes compared to RAF controls, as shown in Figure 1F. To support these observed increases in mitochondrial activity, 24 h after either RAF and COF treatment, live osteochondral explants were stained for ATP (red) and mitochondrial content (blue). COF increased ATP staining relative to all paired RAF controls, as shown in Figure 1G,H. These data demonstrate that CO increases HO1 as well as mitochondrial metabolism in articular chondrocytes rapidly after exposure.

### 3.2. COF Augments Articular Chondrocyte GSH Metabolism and Protects Against Injury

**Figure 2:**
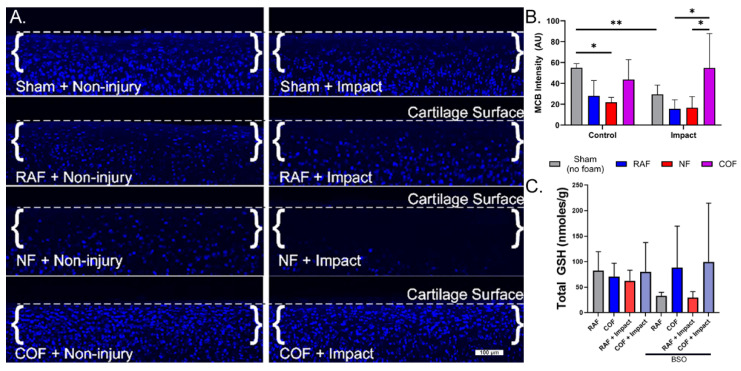
CO augments articular chondrocyte GSH metabolism with and without cartilage injury. (**A**) MCB staining from representative cross-sections of the articular surfaces of foam-treated explants. Osteochondral tissue was impacted with 2 J/cm^2^ impacts immediately after CO or control foam treatments. Control foams caused losses in MCB intensity that suggest redox injury. With impact, control foams did not prevent further loss of MCB staining, while CO prevented oxidation of GSH, indicating the preservation of stain intensity, 10×. (**B**) Quantitative assessment of MCB staining shows COF (magenta)-preserved reduced thiols after foam exposure and injury. (**C**) COF (magenta) increased total GSH compared to RAF (blue) after pretreatment with BSO, depleting GSH; *n* = 4. As determined using Sidak’s multiple comparisons test of two-way ANOVA, * *p* < 0.05, ** *p* < 0.01.

We next evaluated the redox status of articular chondrocytes in situ after COF, RAF, or NF, using live cell staining with MCB in our well-characterized impact model known to incur oxidative stress [10,11,12]. MCB staining from representative cross-sections of the articular surfaces of foam-treated explants are shown in Figure 2A, with dashed lines denoting the cartilage surface in each micrograph. MCB fluorescence intensity decreased by 50% with RAF treatment and 60% with NF treatment, suggesting the PBS-based solutions in the foam (RAF and NF) and hypoxia (NF) caused oxidation in these chondrocytes. This change was comparable to mechanical injury alone, whereas COF maintained MCB staining similar to control, as shown in Figure 2. After injury, MCB fluorescence intensity decreased by 50% with RAF treatment and 44% with NF treatment. However, COF significantly increased the MCB staining intensity of impacted specimens, maintaining reduced thiols intracellularly, as shown in Figure 2B. We only observed cell death in the NF-treated explants, suggesting important distinctions between NF and COF, despite both being constructed without oxygen gas.

To further describe chondrocyte redox changes resulting from CO and explore relationships between CO and GSH, we depleted intracellular GSH with BSO treatment for 24 h prior to injury. Explants were then removed from BSO treatment, given RAF or COF for 1 h, and then subjected to the same impact experiment as above. Though total GSH levels were relatively stable without BSO treatment, after BSO treatment to deplete GSH, COF-treated explants showed significant increases in total GSH content relative to RAF, independent of cartilage injury, as shown in Figure 2C. These data suggest that CO provides support to the GSH pathway in articular chondrocytes.

**Figure 3:**
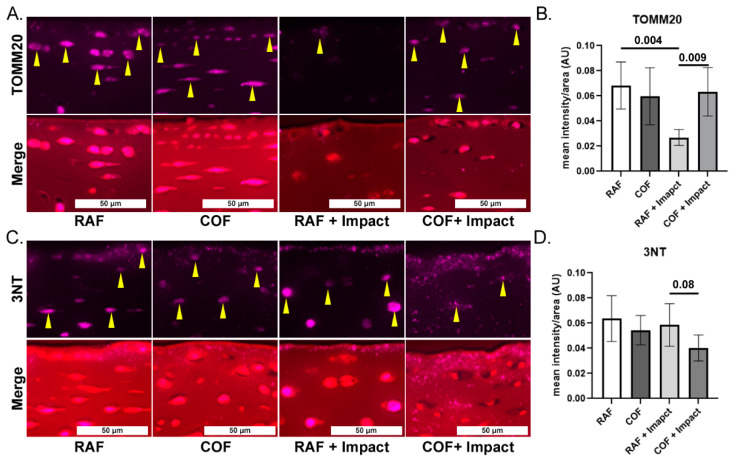
CO pretreatment is mitochondrial protective and decreases oxidative damage after injury. (**A**) Representative images of mitochondrial marker TOMM20 (purple, yellow arrows highlight intracellular staining) and merger with Saf-O, 20×. (**B**) Quantitation of superficial zone demonstrates that RAF + impact significantly decreased TOMM20. The COF + impact had significantly more TOMM20 positivity compared to RAF + impact; *n* = 4, non-parametric Mann–Whitney test. (**C**) Representative images of 3NT staining (purple) and counterstained of articular cartilage with Saf-O (red), 20×. (**D**) Quantitation of 3NT staining demonstrated trend towards decreased 3NT positivity 24 h after COF + impact; *n* = 4, non-parametric Mann–Whitney test.

To evaluate mitochondrial content and link our work to prior studies showing loss of mitochondria after injury, we applied IF staining for mitochondrial marker TOMM20 in fixed tissues and quantified the intensity in the superficial zone. COF treatment prior to impact significantly increased the TOMM20 staining compared to the RAF + impact process, but this was not different compared to COF controls, as shown in Figure 3A,B. To measure oxidation in the superficial zone of these tissues, we applied IF of 3NT-modified proteins to fixed tissues and quantified the intensity of the staining per area. The application of COF prior to impact reduced 3NT formation relative to RAF controls, as shown Figure 3C,D. These data support the hypothesis that CO is stimulating the GSH pathway, protecting chondrocyte mitochondria and decreasing oxidation after injury.

### 3.3. Intra-Articular CO Is Non-Toxic and Increases Reduced Thiols

**Figure 4:**
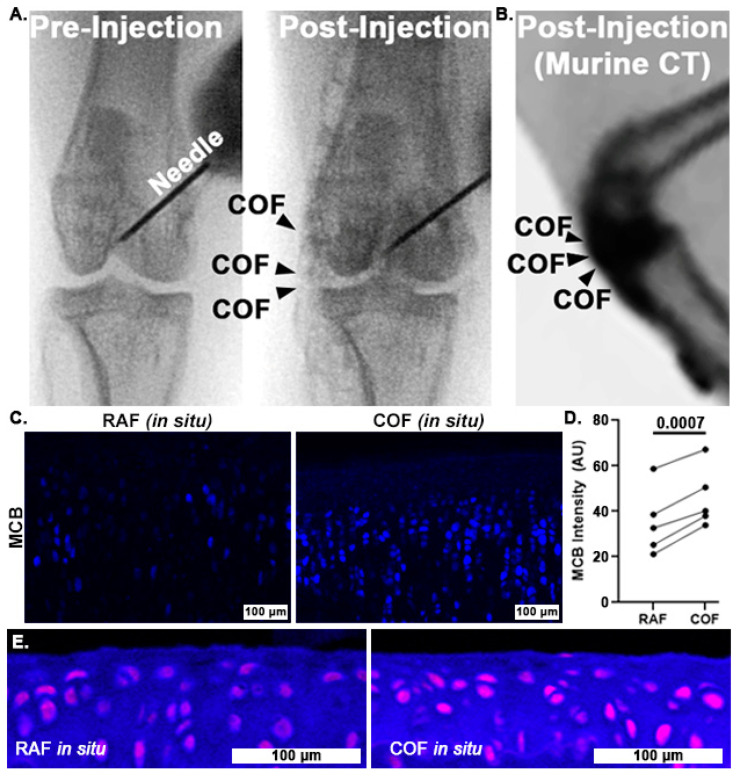
Intra-articular CO is non-toxic to chondrocytes and increases reduced thiols in situ. Fluoroscopy of iodinated COF shows how intra-articular injection can fill stifle joints of rabbits (**A**) and mice (**B**). Rabbit joints exposed to CO in this manner show increased MCB staining compared to contralateral joints exposed to RAF (shown in (**C**), 10×, and quantified in (**D**)). *n* = 5, *p* = 0.0007 by paired *t*-test for MCB analysis. (**E**) Murine articular cartilage stained immunohistochemically for HO1 24 h after foam exposure in situ ((**E**), magenta) was more intense after COF compared to RAF, 10×.

Once we had determined that CO could improve chondrocyte redox and mitochondrial function, we wanted to examine whether COF might reliably deliver CO throughout articular joints and to articular cartilage itself. To accomplish this, we incorporated contrast agent into the foam and injected it into the stifle (quadruped knee) joints of rabbits and mice. Fluoroscopic images of the rabbit stifles after intra-articular COF injection demonstrated that the material filled the joint space, as shown in Figure 4A. We observed signal throughout the stifle, including the articular surfaces as well as superior and distal portions of the joint. Murine CT imaging also showed the retention of the foam within the stifle joint, as shown in Figure 4B. After these in situ exposures, knees were disarticulated and prepared for MCB staining for comparison to bovine explants as well as HO1 staining. MCB staining revealed that cartilage treated with COF exhibited significantly higher reduced thiol signal (30%) compared to paired contralateral joints receiving RAF, *p* = 0.0007, as shown in Figure 4C,D. IF staining with HO1 showed increased staining in the articular cartilage of murine stifles, as shown in Figure 4E.

**Figure 5:**
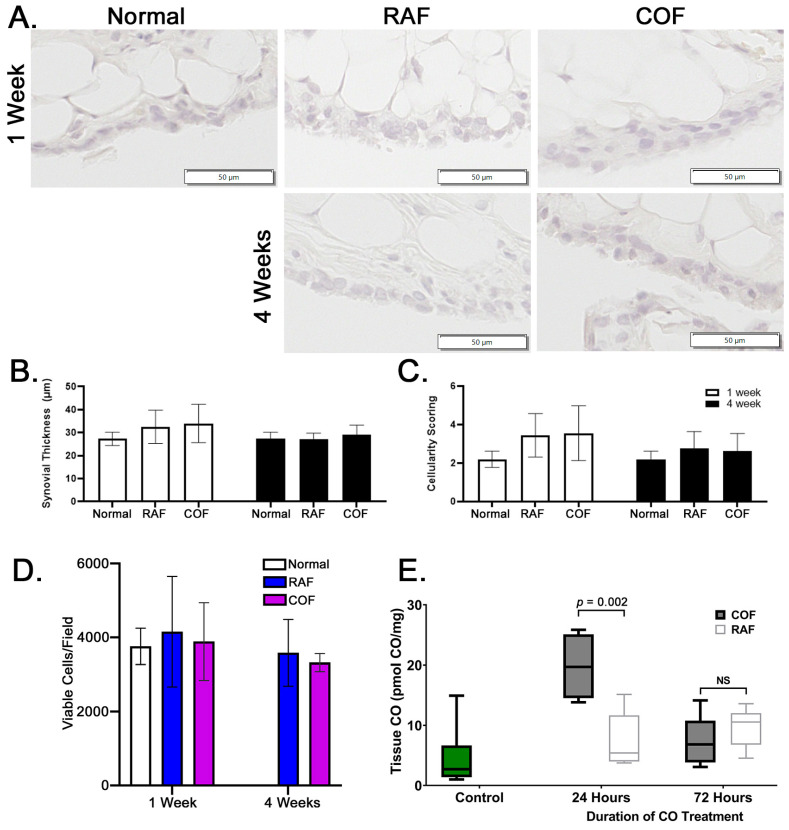
Intra-articular COF injection: non-toxic and retained for 24 h within the joint. (**A**) Representative images of synovia captured from rabbits either 1 week or 4 weeks after COF or RAF injection demonstrate no indications of thickening or increased infiltration by immune cells (quantified in (**B**) and (**C**)). No significant differences were observed in datasets shown, *n* = 10, analyzed via two-way ANOVA, *p* = 0.3 for the effect of treatment. Analyses of live cell densities from rabbits 1 week or 4 weeks after COF or RAF injection demonstrated no indications of any changes in live cell density at either timepoint using viable cell counts per field (**D**). Intact mouse stifle joints were analyzed for CO content 24 and 72 h after injection, *n* = 6 (**E**).

In order to examine whether these injections were safe prior to larger implementation in any species, rabbits were given injections as described above and then allowed normal activity for 1 and 4 weeks prior to euthanasia. After harvest, synovia stained with hematoxylin and eosin were examined for indications of synovial thickening or increased cellularity of the tissue. We observed no indications of thickening or inflammation of the synovium in the rabbits given intra-articular injections of RAF or COF, as shown in Figure 5A–C. In addition, live cartilage showed no changes in articular chondrocyte viability, as indicated by calcein AM positivity; this is shown in Figure 5D, with images shown in Figure S1. These data demonstrated that 1 week after injection and 4 weeks after injection of COF, the tissues of the joint appear normal, viable, and undamaged by the material or treatment. To ensure the delivery of CO was successful and retained in the joint space, murine stifles were examined for CO concentrations using mass spectrometry 24 and 72 h after injection. We observed significant CO remaining in the joint 24 h after injection that did not remain 3 days after injection, as shown in Figure 5E. This demonstrates that significant amounts of CO are retained for at least 24 h after injection.

## 4. Discussion

Mechanical injury of the articular cartilage contributes to PTOA via oxidation and mitochondrial damage [10,11,12]. Mitochondrial abnormalities in chondrocytes are also observed prior to cartilage degeneration in animal models of PTOA and these pathways contribute to disease progression [41,42,43]. This has led to enthusiasm within the orthopedic community for enhancing mitochondrial function and reducing redox stress. In this study, we show the direct effects of CO on articular cartilage redox status and mitochondria. CO rapidly induced HO1, increased mitochondrial function, and improved redox status in large-animal tissue. CO also protected chondrocyte mitochondria and redox status against mechanical injury and was well tolerated in vivo, incurring no toxicity or inflammation.

These data demonstrate the acute, protective effects of CO on articular cartilage, which may be efficacious for mitigating injury to articular cartilage. Though preventive opportunities to provide clinical care are rare, we envision COF application prior to any appropriate orthopedic procedure where cartilage protection is of interest and exposed to the rigors of surgery. Our treatment scheme in this study also allows for scientific comparison to prior agents that manipulate mitochondrial or GSH pathways with pretreatment or cotreatment with impact or injury [10,11,12]. We observed comparable effects between COF and prior antioxidants tested (N-acetylcysteine, tempol, tocopherol, superoxide dismutase mimetics) in terms of GSH oxidation, but consistently observed increases in mitochondrial readouts with COF that were noteworthy. We previously observed these increases with tempol or tocopherol, which may suggest a linkage between the biliverdin liberated by CO and other lipid antioxidants [12]. Future examinations of COF will continue to explore how mitochondrial metabolism and specific reactive species are linked.

To add rigor to our study, we included in vitro large-animal explant models, ex vivo models of rabbit and mouse stifle joints, and in vivo animal models that each showed consistent effects upon mitochondria and GSH when CO was introduced to articular cartilage. We adopted multiple approaches for investigating mitochondria, including the expression of pro-mitochondrial pathways and activity of live mitochondria as soon as possible after CO exposure. Because CO induces HO1 and augments mitochondrial respiration within a short-time frame, we concentrate primarily on the effects of CO in the most superficial zone of tissue where dosing and injury are most predictable. The delivery of CO from COF relies on diffusion-, depth- and time-specific effects and we expect this will be important for future preclinical and translational efforts. More detailed depth- and diffusion-focused studies could provide interesting kinetic views of how CO’s chemical activity alters short-term chondrocyte metabolic behavior. Large-animal models with similar cartilage thickness to humans will be used in future studies to evaluate these time-dependent and dose-dependent effects of gasosignaling in intra-articular biology.

One feature of these studies that surprised us was the durability of effects from the CO exposure. The effects of CO persisted for 24 h after exposure and COF-treated chondrocytes were able to rapidly overcome the BSO blockade of GSH synthesis. We propose that the direct benefits of CO (i.e., more functional chondrocyte mitochondria, robust antioxidant defenses, and the induction of HO1) are ideally suited to post-injury and surgical repair procedures that may expose joints to stress for extended periods of time.

## 5. Conclusions

We have shown here how COF is easily injectable into rabbit and mouse joints; thus, it represents a realistic solution for human intra-articular injection. COF also consists of simple, generally recognized as safe (GRAS) materials which confer meaningful, lasting safety and biocompatibility. Thus, COF is an ideal adjuvant to orthopedic surgical care in any context, where damage to cartilage is a concern. In the long term, we expect the local application of CO to protect against the development of PTOA, not only by coordinating chondrocyte intracellular metabolism, but also by coordinating related pro-inflammatory/anti-inflammatory signaling throughout the joint.

## Data Availability

All data created for this manuscript are reported herein and can be made available upon request.

## Supplementary Materials

The following supporting information can be downloaded at: https://www.mdpi.com/article/10.3390/antiox14050514/s1, Figure S1: Intra-articular Administration of COF Causes no Toxicity. We observed no decreases in viable cell density in any groups at either 1 week or 4 weeks after injection compared to normal cartilage. Images are taken from rabbit tibias and the irregular appearance of the staining is the so-called line artifact created by the confocal scanning and subsequent Z-stacking of the scanned images of a curved surface.
