# Supplementary figures and images for: Carbon Monoxide Stimulates Chondrocyte Mitochondria and Protects Mitochondria During Cartilage Injury
Source: Antioxidants (Basel). 2025 Apr 25;14(5):514. doi: 10.3390/antiox14050514 (PMC12108337; doi:10.3390/antiox14050514)

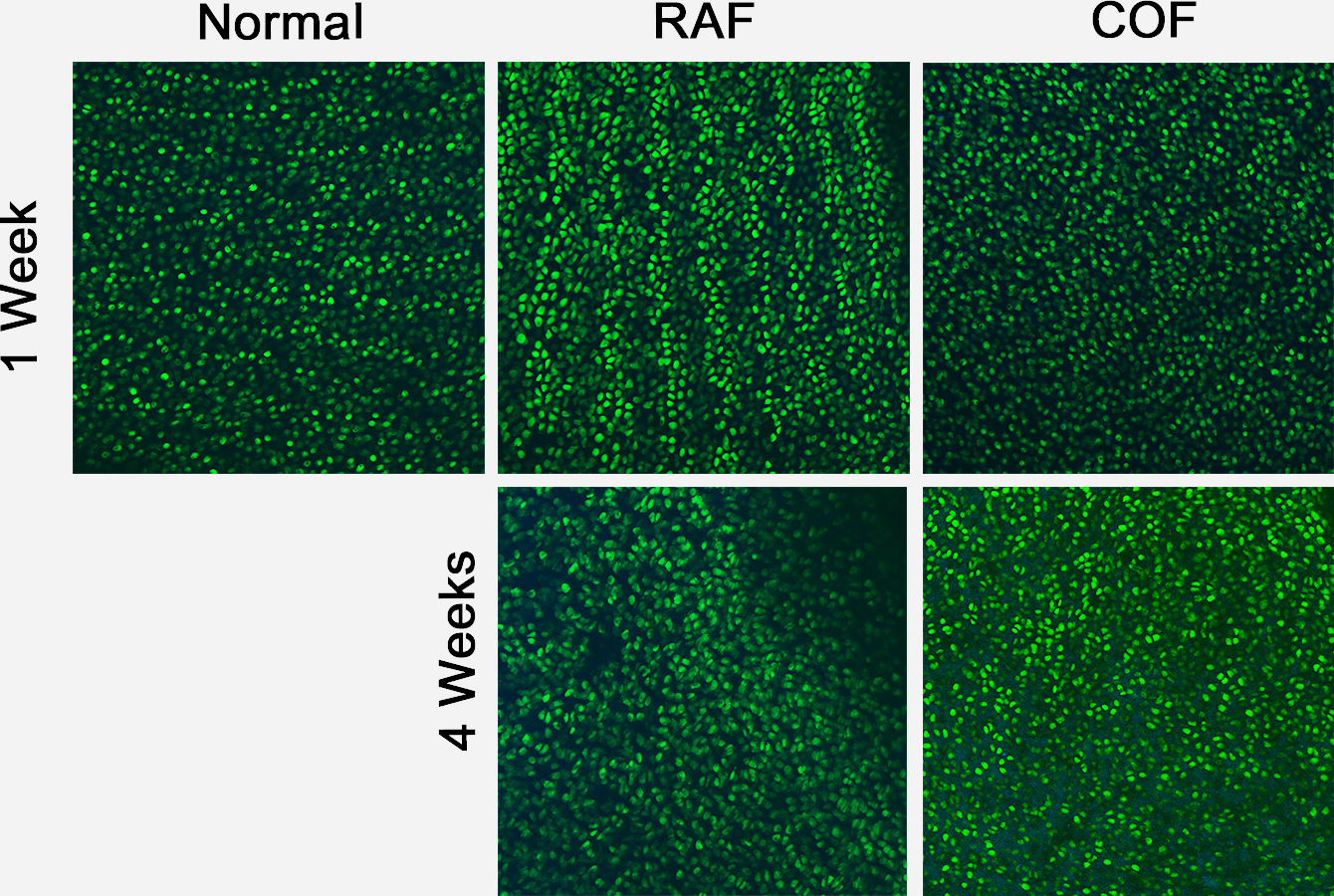

Supplement: Supplementary file 1 [file antioxidants-14-00514-s001.zip › antioxidants-3575790-supplementary.tif]
